# Local Gene Expression Changes after UV-Irradiation of Human Skin

**DOI:** 10.1371/journal.pone.0039411

**Published:** 2012-06-22

**Authors:** Benjamin Weinkauf, Roman Rukwied, Hans Quiding, Leif Dahllund, Patrick Johansson, Martin Schmelz

**Affiliations:** 1 Department of Anesthesiology and Operative Intensive Care, Medical Faculty Mannheim, University of Heidelberg, Mannheim, Germany; 2 AstraZeneca R&D, Södertälje, Sweden; University of Tokyo, Japan

## Abstract

UV-irradiation is a well-known translational pain model inducing local inflammation and primary hyperalgesia. The mediators and receptor proteins specifically contributing to mechanical or heat hyperalgesia are still unclear. Therefore, we irradiated buttock skin of humans (n = 16) with 5-fold MED of UV-C and assessed the time course of hyperalgesia and axon reflex erythema. In parallel, we took skin biopsies at 3, 6 and 24 h after UVC irradiation and assessed gene expression levels (RT-PCR ) of neurotrophins (e.g. NGF, BDNF, GDNF), ion channels (e.g. NaV1.7, TRPV1), inflammatory mediators (e.g. CCL-2, CCL-3) and enzymes (e.g. PGES, COX2). Hyperalgesia to mechanical impact (12 m/s) and heat (48°C) stimuli was significant at 6 h (p<0.05 and p<0.01) and 24 h (p<0.005 and p<0.01) after irradiation. Axon reflex erythema upon mechanical and thermal stimuli was significantly increased 3 h after irradiation and particularly strong at 6 h. A significant modulation of 9 genes was found post UV-C irradiation, including NGF (3, 6, 24 h), TrkA (6, 24 h), artemin, bradykinin-1 receptor, COX-2, CCL-2 and CCL-3 (3 and 6 h each). A significant down-regulation was observed for TRPV1 and iNOS (6, 24 h). Individual one-to-one correlation analysis of hyperalgesia and gene expression revealed that changes of Nav1.7 (SCN9A) mRNA levels at 6 and 24 h correlated to the intensity of mechanical hyperalgesia recorded at 24 h post UV-irradiation (Pearson r: 0.57, p<0.04 and r: 0.82, p<0.001). Expression of COX-2 and mPGES at 6 h correlated to the intensity of heat-induced erythema 24 h post UV (r: 0.57, p<0.05 for COX-2 and r: 0.83, p<0.001 for PGES). The individual correlation analyses of functional readouts (erythema and pain response) with local expression changes provided evidence for a potential role of Nav1.7 in mechanical hyperalgesia.

## Introduction

The UV-irradiation has extensively been used as a translational model for inflammatory pain and hyperalgesia including studies in rodents [1]–[4], pigs [5] and human volunteers [6]–[10]. The time course of hyperalgesia development is similar in different species, with an onset latency of 3–6hours and peak responsiveness 24–48 hours after irradiation, thus representing a useful experimental model for drug testing [11], [12].

A multitude of mediators are being released upon UV-irradiation of the skin, including eicosanoids (e.g. PGE2, PGD2, PGF2a, LTB-4, 12-HETE), cytokines (e.g. IL-1, IL-6, IL-8, TNF-alpha), growth factors (e.g. TGF-beta, VEGF, NGF) vasoactive amines and neuropeptides (e.g. histamine, bradykinin, CGRP) (for review see e.g. [13]). Some of these can be accounted for the inflammatory UV-induced responses, such as erythema (i.e. CGRP) or heat hyperalgesia. The latter can be explained by acute sensitization of nociceptors by various mediators (e.g. PGE2, bradykinin) and sensitization of heat-sensing ion channels (e.g. TRPV1). Another cardinal symptom of UV-inflammation in human skin is a profound peripheral mechanical sensitization. The mediators contributing to this phenomenon are largely unknown as acute application of e.g. PGE2, bradykinin or CGRP does not provoke mechanical hyperalgesia in human skin. There is recent evidence for a role of mechanical sensitization of heat insensitive (CM) fibers after UV-irradiation to particularly strong mechanical stimuli [3]. This finding indicates that altered encoding properties of nociceptors could explain mechanical hyperalgesia. However, the contributing receptor proteins or axonal ion channels involved in mechanical hyperalgesia are still unclear.

Here, we investigate which mediators and receptor proteins are being up-regulated during hyperalgesia development. Mechanical and heat hyperalgesia was induced by irradiation with 5-fold minimum erythema dose (MED) of UV-C in volunteers. In contrast to the well-established UV-B model, the UV-C irradiation has been chosen as its shorter wavelength penetrates human skin only very superficially and is entirely absorbed by the epidermis, causing only a very mild sunburn. Thereby, we intended to induce hyperalgesia at a lower inflammatory level as compared to UV-B. Changes of protein expression under these conditions therefore would be expected to be closer linked to hyperalgesia. Following UV-C irradiation and assessment of hyperalgesia, skin biopsies were obtained from these test sites and expression patterns of inflammatory mediators, receptor proteins and ion channels, respectively, were analysed. A selection of candidate genes that might be related to hyperalgesia were screened. Apart from total gene expression changes we additionally correlated the fold-changes of expression to the relative increase of mechanical or heat-induced pain and erythema. Thereby, we aimed to more specifically identify those targets among the pro-inflammatory factors that seem to be of particular relevance for the induction of inflammatory mechanical and heat hyperalgesia.

## Results

No side effects of the irradiation such as blisters, infection or scarring were observed. Moreover, a lasting pigmentation as regularly reported after 3-fold MED UV-B irradiation was not observed upon UV-C exposure. Among the individuals, no correlation between the individual UV-C irradiation dose required for the 5-fold MED, and the mechanical or thermal hyperalgesia was determined (r < −0.4, data not shown).

### Mechanical Sensitization

Stimulation with 14 mN Semmes Weinstein monofilament did not evoke significantly different sensations between the control and UV-C irradiated sites at 24 h post treatment (NRS 7±1 (UV-C and control site), p>0.9, Wilcoxon matched pairs test). Supra-threshold pin-prick stimuli of 512 mN also failed to induce significantly elevated pain in UV-C skin (VAS 32±1 vs. 29±3 (control), p>0.07, Wilcoxon matched pairs test).

**Figure 1:**
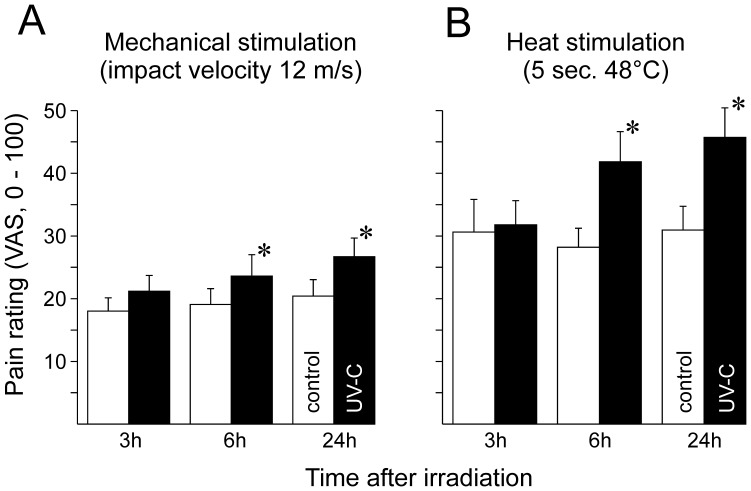
Pain ratings (VAS, 0–100) upon (A) mechanical and (B) heat stimuli. Mechanical impact stimuli were delivered at 12 m/s and continuous heat applied for 5 sec at 48°C. Responses recorded at 3, 6 and 24 h post irradiation are depicted in open columns from untreated skin and those recorded from UV-C treated skin (5-fold MED) in black columns. Asterisks indicate significant differences between control and UV-C skin (p<0.01, Fishers LSD test).

Dynamic mechanical stimuli delivered at an impact velocity of 12 m/s induced a pain response of VAS 19±3 on average in control skin (fig. 1 A). Significantly enhanced pain was recorded at the UV-C treated sites (p<0.01, ANOVA), after 6 h (VAS 24±3, p<0.03, Fisher’s LSD test) and even more after 24 h (VAS 27±3, p<0.005, LSD test).

### Heat Sensitization

Continuous contact heat stimuli induced pain ratings of 30±4 (VAS) at the control sites, which was not significantly different 3 h after UV-C exposure (VAS 32±4, p>0.8, LSD test). In contrast, heat-evoked pain increased 6 h (VAS 42±5, p<0.01) and 24 h (VAS 46±5, p<0.01) after UV-C irradiation (fig. 1 B). Similarly, the area under the curve (AUC) of heat pain ratings was significantly increased at the UV-C sites at these times (p<0.005, data not shown).

### Erythema Responses

**Figure 2:**
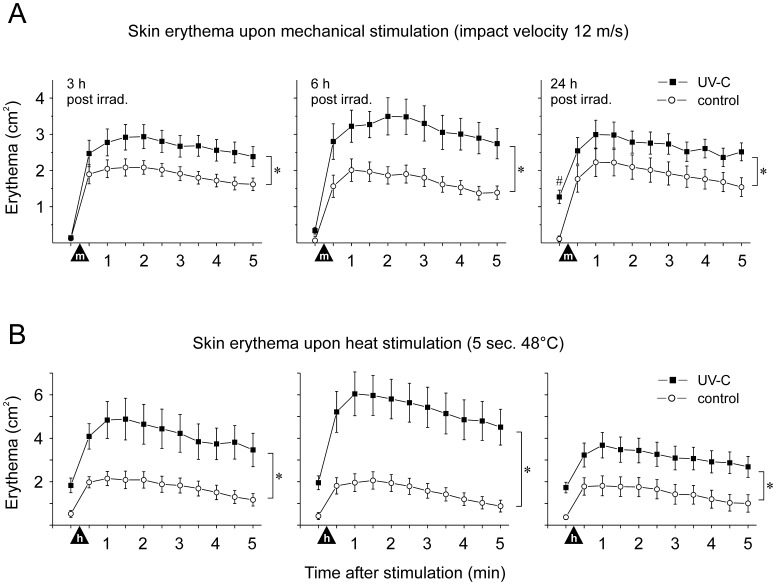
Time course of skin erythema development (cm^2^) upon (A) mechanical and (B) heat stimuli. Responses to mechanical impact stimuli (12 m/s) and continuous heat (5 sec at 48 C) were recorded from untreated control (open circles) and UV-C treated skin (5-fold MED, solid squares) at 3 h (left), 6 h (middle) and 24 h (right panel) post irradiation. The hatch indicates significantly increased blood flow 24 h after UV-C irradiation (p<0.0001, Fishers LSD test), filled triangles indicate the time of stimulation (m: mechanical, h: heat), and asterisks indicate significant differences between control and UV-C skin (p<0.0001, Fishers LSD test). Note that heat tests were performed post mechanical stimulation that had caused slightly increased baseline perfusion.

In comparison to the control skin sites, UV-C irradiation did not significantly change baseline skin blood flow at 3 and 6 h, whereas a localized skin erythema of 1.3±0.2 cm^2^ developed at 24 h post treatment (p<0.0001, LSD test) (fig. 2 A, right panel).

Mechanical impact stimuli delivered at 12 m/s elicited an erythema of about 2±0.3 cm^2^ within 1–2 min as a consequence of the local noxious stimulation apparently causing the release of inflammatory mediators. UV-C irradiation significantly increased mechanically induced erythema responses to 2.8±0.3 cm^2^ at 3 h and to about 3±0.4 cm^2^ at 6 and 24 h (p<0.0001, LSD test) (fig. 2 A).

Supra-threshold heat stimulation (48°C, 5 s) caused an erythema of about 1.9±0.4 cm^2^ in control skin (fig. 2 B). At the UV-C treated sites, heat-evoked erythema responses significantly increased after 3 h to about 4.6±0.8 cm^2^, reached a maximum of 5.7±0.9 cm^2^ at 6 h and declined at 24 h to 3.4±0.6 cm^2^ (all time points p<0.0001, LSD test) (fig. 2 B).

### Expression Patterns

**Figure 3:**
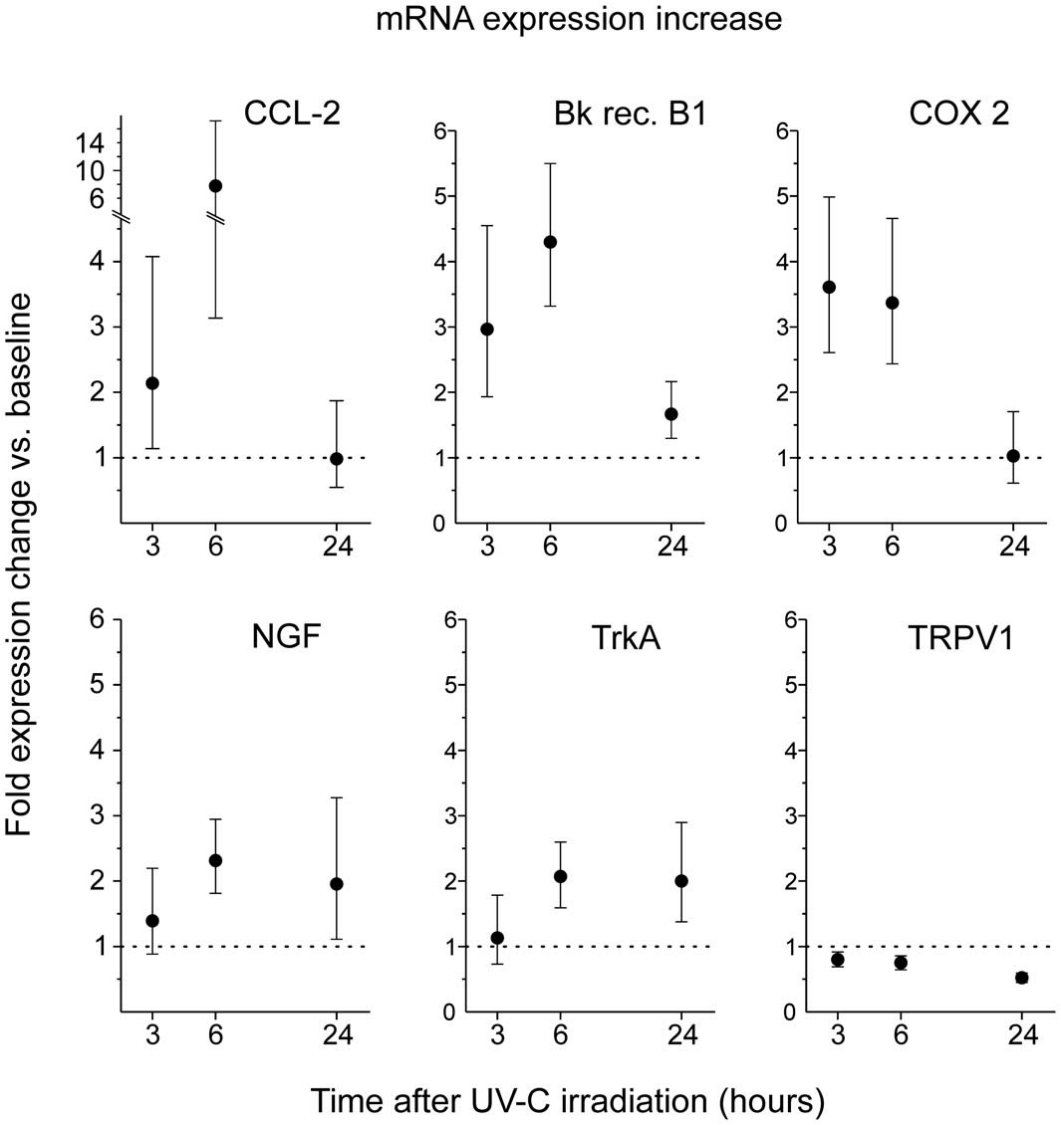
Group analysis of mRNA expression increase (RT-PCR, n = 14). Changes upon UV-C irradiation are depicted as fold increase above baseline (dashed horizontal line represents no change) assessed at 3, 6 and 24 h post irradiation. Error bars indicate the 95% confidence interval.

In the RT- PCR analysis, the expression patterns of 31 genes were investigated and 23 out of those genes could be reliably quantified (table 1). Group analysis revealed an up-regulation from baseline upon UV-C irradiation for artemin, bradykinin-receptor 1 (B1), chemokine ligands CCL-2 and CCL-3, chemokine receptor CCR2, the cyclooxygenase COX-2, nerve growth factor NGF and its high-affinity receptor TrkA (fig. 3, table 1). A large number of genes were expressed, but not regulated, for example sortilin, prostaglandin E synthase (mPGES), the protease-activated receptor 2 (PAR-2), the low affinity neurotrophin receptor p75, the potassium-channel TREK-1, or the gene SCN9A that encodes the sodium channel NaV1.7. mRNA for inducible nitric oxide synthase (iNOS) and the transient receptor potential vanilloid 1 channel (TRPV1) was down-regulated.

**Table 1:** Compilation of relative gene expression 3, 6 and 24 h after UV-C irradiation (n = 14).

Gene	protein	expression 3 h	expression 6 h	expression 24 h
CCL2**	monocyte chemotactic protein, MCP1	**2.17 (1.1–4.5)**	**7.39 (3.2–16.9)**	1.03 (0.6–1.8)
CCL3***	macrophage inflam. protein 1- α	**3.03 (1.1–8.0)**	**5.08 (2.2–12.0)**	0.49 (0.1–1.6)
BDNF*	brain derived neurotrophic factor	1.97 (0.7–5.5)	**2.67 (1.2–6.2)**	1.42 (0.6–3.3)
BDKRB1***	bradykinin B1 receptor	**2.98 (1.9–4.6)**	**4.29 (3.3–5.5)**	**1.68 (1.3–2.2)**
PTGS2***	cyclooxygenase 2, COX-2	**3.60 (2.6–4.9)**	**3.37 (2.4–4.7)**	1.03 (0.6–1.7)
NGF*	nerve growth factor	1.39 (0.9–2.2)	**2.31 (1.8–3.0)**	**1.96 (1.1–3.3)**
NTRK1***	high affinity NGF receptor, trkA	1.13 (0.7–1.8)	**2.08 (1.6–2.6)**	**2.00 (1.4–2.8)**
ARTN***	artemin	1.71 (1.2–2.4)	**1.91 (1.5–2.4)**	1.27 (1.0–1.6)
NGFR*	low affinity receptor, p75	1.42 (0.9–2.3)	**1.63 (1.1–2.3)**	1.15 (0.9–1.4)
MPO	myeloperoxidase	1.24 (0.8–1.9)	1.27 (0.8–2.0)	1.11 (0.7–1.8)
CCR2***	chemokine receptor 2	0.82 (0.6–1.1)	1.27 (0.9–1.8)	**1.86 (1.3–2.6)**
GDNF**	glial cell derived neurotrophic factor	1.14 (0.8–1.7)	1.31 (1.0–1.8)	**1.74 (1.1–2.8)**
GFRA3	GDNF family receptor α 3	1.29 (1.0–1.7)	1.19 (0.9–1.5)	0.99 (0.7–1.3)
KCNK2	two pore domain K^+^ channel, TREK-1	1.12 (0.9–1.4)	1.16 (0.9–1.5)	1.10 (0.9–1.4)
SCN3A*	voltage gated sodium channel, Nav1.3	1.06 (0.9–1.3)	1.17 (1.0–1.4)	0.68 (0.6–0.8)
GFRA1*	GDNF family receptor α 1	1.04 (0.9–1.1)	1.09 (0.9–1.3)	0.85 (0.7–1.1)
F2RL1	proteinease actived receptor, PAR2	1.11 (1.0–1.3)	1.12 (1.0–1.3)	0.91 (0.7–1.1)
SCN9A	voltage gated sodium channel, NaV1.7	1.23 (1.0–1.5)	1.05 (0.9–1.2)	0.92 (0.8–1.1)
PTGES	prostaglandin E synthase	0.93 (0.8–1.0)	0.97 (0.8–1.1)	**1.25 (1.1–1.5)**
NOS2*	nitric oxide synthetase 2	**0.72 (0.5–1.1)**	0.63 (0.4–1.0)	**0.52 (0.3–0.9)**
SORT1	sortilin	**0.85 (0.7–1.1)**	0.79 (0.6–1.0)	0.83 (0.6–1.1)
TRPV1***	transient receptor potential cation channel 1	**0.80 (0.7–0.9)**	**0.75 (0.7–0.9)**	**0.52 (0.5–0.6)**
PTGS1***	cyclooxygenase 1, COX-1	**0.76 (0.7–0.9)**	**0.73 (0.7–0.8)**	0.95 (0.9–1.0)

Values depict ratios to baseline condition (geometric mean and 95% confidence interval, bold face indicates means outside the 95% confidence interval). (*, **, ***: p<0.05, 0.01, 0.001; repeated measures ANOVA for 3, 6 and 24 h expression level for each gene).

### Correlation of Gene expression and Functional Data

**Figure 4:**
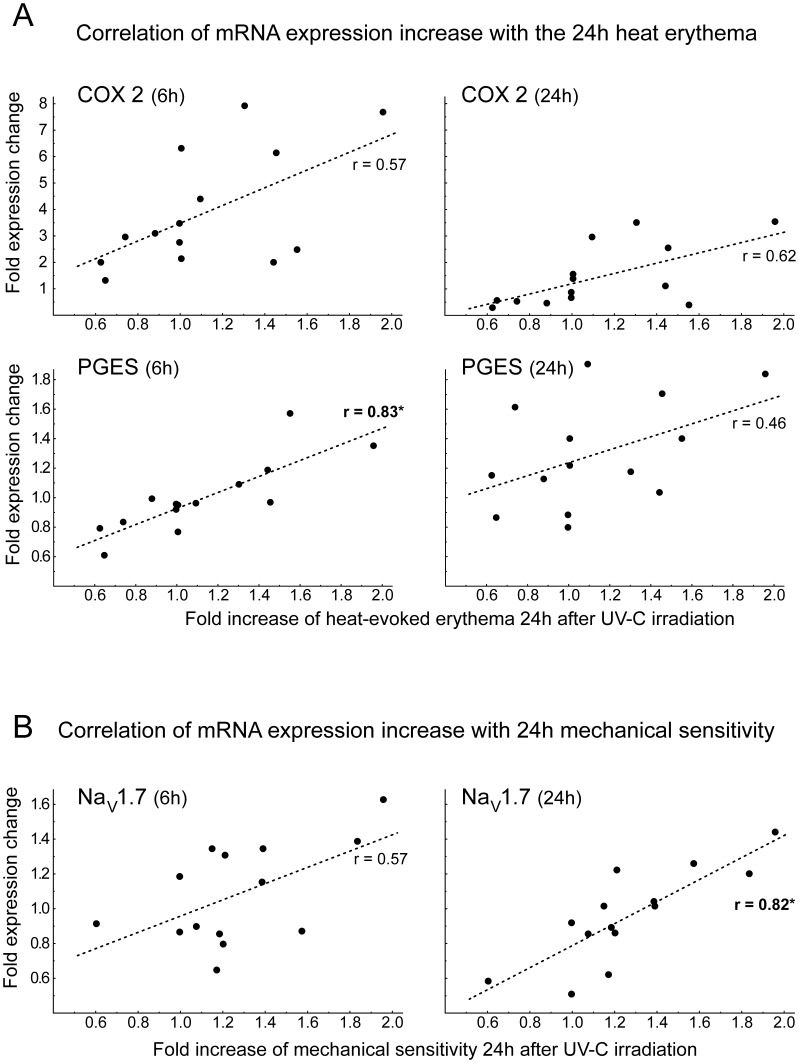
Correlation analysis of mRNA expression (n = 14) and 24 h hypersensitivity upon (A) heat and (B) mechanical impact. Individual one-to-one correlation analyses of functional hypersensitivity 24 h post UV-C irradiation and mRNA expression changes at 6 h (left panel) and 24 h (right panel). All values were normalized to control skin. (**A**): Correlation of relative increase of cyclooxygenase 2 (COX-2, upper panels) and prostaglandin synthase (mPGES, middle panels) expression with relative increase of heat-evoked erythema (48°C, 5 sec) recorded at 24 h after irradiation. (**B**): Correlation of NaV1.7 (gene SCN9A) expression changes at 6 h (left) and 24 h (right) after UV-C irradiation with relative increase of mechanical impact pain (12 m/s) recorded 24 h post irradiation. Significant correlations (p<0.05, Bonferroni corrected) are marked by asterisks.

An intra-individual one-to-one correlation analysis was performed for the erythema and hyperalgesia recorded at 24 h after UV-C irradiation with the 6 and 24 h gene expression changes. A positive correlation was identified for up-regulated COX-2 at 6 and 24 h after UV-C irradiation and an increased heat evoked erythema at 24 h (Pearsons r: 0.57; (6 h) and r:0.62; (24 h)) but after Bonferroni correction no significant difference was detected (fig. 4A). Prostaglandin E synthase (mPGES) correlated significantly at 6 h (r: 0.83, p<0.03, Bonferroni corrected), but not at 24 h (r: 0.46), to the intensity of the UV-C induced erythema after heat stimulation at 24 h (fig. 4 A). Interestingly, regulation of the gene encoding Nav1.7 (SCN9A) appeared to correlate at 6 h (Pearson r: 0.57) and was significant at 24 h (r: 0.82, p<0.05, Bonferroni corrected) after irradiation to the intensity of peak mechanical hyperalgesia at 24 h (fig. 4 B).

## Discussion

UV-C skin irradiation with 5 fold MED causes a mild hyperalgesia to heat and dynamic mechanical impact stimuli, which developed 6 h post irradiation and sustained for at least 24 h. Noteworthy, UV-C evoked sensitization was not as strong as upon 3-fold UV-B irradiation [6], the well-established model of profound inflammatory hyperalgesia [3], [7], [8], and therefore, identified gene expression pattern changes after UV-C irradiation would represent sensitive markers for targets of hyperalgesia. A modulated gene expression pattern was found in the irradiated skin for e.g. bradykinin receptor 1, chemokine ligand CCL-2, COX-2, NGF and its high affinity receptor TrkA. Analyzing the individual pain responses upon heat and mechanical stimuli with the individual mRNA expression patterns, we found a correlation between COX-2 and PGES levels at 6 h and heat evoked erythema, and identified in addition a possible role of Nav1.7 (gene SCN9A) for mechanical hyperalgesia.

### Time Course of Hyperalgesia

UV-C irradiation caused a delayed local reddening at 24 h post irradiation. Hyperalgesia to heat and mechanical stimuli developed already 6 h after UV-C treatment and was sustained for 24 h. Considering that UV-C affects the uppermost superficial skin layers and is completely absorbed by the epidermis, keratinocytes are the primary target of the irradiation. They synthesize and release a variety of inflammatory mediators upon irradiation [13] which can directly sensitize intraepidermal branches of nociceptors. In contrast, blood vessels are located intradermally and thus, vasodilation is not necessarily linked to nociceptor sensitization. This mismatch between vasodilation and nociceptor sensitization has been shown, for instance, after administration of NGF, which causes an impressive nociceptor sensitization but does not induce any vasodilation [14]. As shown previously, axon reflex erythema is mediated by mechano-insensitive C-fibres [15], a nociceptor sub-population that become sensitized after UV-B irradiation [16]. Accordingly, not only supra-threshold heat but also mechanical impact stimuli caused an elevated erythema response in UV-C treated skin. However, also in normal skin noxious mechanical impact stimuli have been reported to provoke axon reflex and even plasma extravasation [17], indicating cell damage. Thus, chemical activation of nociceptors also may have contributed to the axon reflex following strong impact stimulation.

UV-B irradiation is known to recruit as variety of immune cells, such as neutrophils, macrophages and lymphozytes [18], [19]. Thus, it may be assumed that these inflammatory cells are also involved in the UV-C induced hyperalgesic responses and, in addition, are most likely contributing to altered gene expression. This issue should be investigated in the future by focussing on specific cell markers, but also by separately analysing epidermal and dermal expression patterns.

### Expression Analysis

Among inflammatory mediators, the identified bradykinin receptor 1 up-regulation particularly at 6 h after UV-C exposure provides an interesting functional evidence for increased sensitivity in human sunburn. In the rat, thermal hyperalgesia after UV-irradiation appears predominantly mediated via B1 receptors [20]. An increased B1 and B2 receptor mediated hypersensitivity as well as enhanced local vasodilatation upon B1-receptor activation has been described in UV-B irradiated human skin before [9]. Here, 24 h after UV-C sensitization, we found a strong correlation for mechanically and thermally induced blood flow increases and an up-regulation of B1 receptors, suggesting that experimentally evoked erythema might be attributed, at least partially, to B1 receptors. In addition, it was shown previously that bradykinin induced thermal hyperalgesia is mediated by cyclooxygenase products [21]. Indeed, following UV-C exposure, COX-2 was up-regulated and this is in-line with the well-known anti-inflammatory and analgesic effect of COX-2 inhibitors in human sunburn [22].

Amongst cytokines, we identified a strong increase of the chemokine (C-C motif) ligand 2 (CCL-2) in UV-C skin, which is in accordance with a recent study exploring the cytokine profile after UV-B irradiation in human skin [18]. Apart from the extremely up-regulated chemokine (C-X-C motif) ligand CXCL-5, which we unfortunately did not analyse in our samples, the authors analysed a 5-fold increase of CCL-2 [18] matching the 7-fold transcript level increase of CCL-2 found in our study. Interestingly, CCL-2 induced mechanical and thermal hyperalgesia upon intradermal injection [23] and its up-regulation correlated to postoperative pain [24].

Apart from cytokines, growth factors were already hypothesized to participate in UV-induced nociceptor sensitization. Of these, nerve growth factor (NGF) and its high affinity receptor TrkA are of particular interest as it induces local thermal and mechanical hyperalgesia in rodents [25] and humans [14], [26] upon injection in the skin. Importantly, sequestering NGF after UV-B irradiation significantly reduced mechanical and thermal hypersensitivity [4] suggesting a major role of NGF for UV-induced nociceptor sensitization. NGF levels increase following UV stimulation, which has been shown on protein [1] and mRNA level [27] in rodents. Albeit our data strongly support UV-induced up-regulation of both NGF and TrkA, there are also reports on reduced NGF levels in human keratinocytes and melanocytes [28] and reduced dermal TrkA immunostaining [29] after UV-irradiation. Thus, based on these studies, no precise conclusions should be drawn on the impact of NGF and TrkA in UV-evoked heat hyperalgesia.

We did not observe an up-regulation of TRPV1 after UV-irradiation, which appears to be in contrast to the observed hyperalgesia to heat. However, it can be assumed that any regulation is mainly based on the keratinocyte expression as these are primarily affected by the UV-C irradiation. Albeit there is evidence for local protein biosynthesis in dendrites and axons [30], potential expression changes of axonal mRNA would involve signal transport from the irradiated skin to the dorsal root ganglion (DRG), induction of expression changes and subsequent trafficking of mRNA back to the skin. Considering these events, TRPV1 expression changes at 6 and 24 h after irradiation would be expected too early. Also, if there were axonal mRNA alterations, these would not necessarily contribute to expression changes due to the massive dilution of RNA caused by resident or infiltrating cells. Moreover, our data do not contradict a role of TRPV1 underlying heat hyperalgesia, particularly as heat sensitization, for instance by TRPV1 phosphorylation or TRPV1 translocation in the sensory endings, obviously does not require expression changes.

### Correlation between Individual Functional Read Outs and Expression Changes

Parallel time courses of the pooled expression change and of the inflammatory response could be indicators for a functional link. Even more specifically, a link between expression changes and functional read out could be drawn when analyzing the individual data at given time points. We therefore correlated the expression changes assessed from each subject 6 and 24 h after the irradiation with his increase of erythema upon nociceptor activation, as well as with his hyperalgesic pain response upon mechanical and heat stimuli, recorded at 24 h after irradiation.

For COX-2, which was upregulated 3 and 6 h after the UV-C irradiation, we identified a positive correlation between increased expression at 6 h and increased heat-evoked erythema 24 h after irradiation. The heat-evoked erythema intensity also correlated to the PGES expression change (see fig. 4), a finding which is rather unexpected considering that no significant regulation for PGES was found in the pooled expression analysis (see tab. 1). Apparently, group analysis did not detect a significant regulation of this enzyme, whereas individual data correlation uncovered those subjects with a particular strong erythema response having up-regulated PGES. Regardless of the potential functional role of PGES for increased heat-evoked erythema in the UV burn, our results suggest that the individual correlation analysis between functional parameters and expression changes provide independent additional information as compared to group analyses.

An individual one-to-one analysis might be even more important for exploring specific mediators or receptors linked to inflammatory hyperalgesia - it could be grossly assumed that the degree of inflammation correlates to the degree of nociceptor sensitization. This assumption, however, is not mirrored in osteoarthritis, in which anti-NGF therapy has been shown to provide analgesia that was not accompanied by anti-inflammatory effects [31]. Thus, pro-inflammatory effects and nociceptor sensitization apparently do not necessarily correspond. Correlating UV-induced expression changes with individual nociceptor sensitization and the degree of inflammatory responses might therefore be a promising approach to reveal a possible functional relation.

Interestingly, an expression that correlated best to mechanical hyperalgesia was found for the NaV1.7 gene SCN9A. Again, as observed for PGES, this is rather surprising as there was no significant regulation by UV in the group analysis for this axonal ion-channel. Interestingly, sodium channel expression in keratinocytes has been linked to neuropathic pain [32] with pain patients showing increased keratinocyte staining for NaV1.5, 1.6 and 1.7. Thus, not only neuronal but also keratinocyte NaV1.7 might contribute to mechanical hyperalgesia. However, verification of altered protein levels and functional consequences are required to substantiate this potential link. Moreover, a further validation of this correlation is warranted using a more pronounced hyperalgesia induced by UV-B.

In summary, we analysed UV-induced gene expression changes in correlation with the individual read-outs of inflammation and nociceptor sensitization in human volunteers. Group analysis of total mRNA transcripts confirmed the increased expression of various pain- and inflammation-related targets, such as bradykinin B1 receptor and COX-2. Individual data correlation revealed a possible link between COX-2 and PGES to heat-evoked erythema response. In addition, the expression of NaV1.7 correlated to mechanical hyperalgesia. Noteworthy, neither PGES nor NaV1.7 were regulated in the group analysis. We conclude that in this model of mild inflammatory hyperalgesia the correlation between individual expression and sensory changes represents a promising approach to differentiate the impact of regulated genes on inflammation and nociceptor sensitization.

## Materials and Methods

The study protocol has been approved by the local Ethics Committee II of the Medical Faculty Mannheim. Sixteen healthy male subjects (average age 33.9±11.5 yrs), Fitzpatrick skin type II–III, were enrolled after having given their written informed consent. All volunteers attended a training session to familiarize with the experimental procedure of pain ratings recorded with visual analogue scale (VAS) upon dynamic mechanical impact stimulation and the quantitative sensory test (QST) upon localized heat application.

### UV-C Irradiation

During the training session, the lateral sites of the left and right buttock of each panellist were irradiated with ascending UV-C doses (9–13–23–33–42–59–64–91 mJ) delivered to a 1 cm^2^ skin area by means of a calibrated UV-C source (SENSELab, Somedic, Hörby, Sweden) in order to determine the minimal erythema dose (MED) required to induce a sunburn.

After 24 h, the irradiated sites were investigated and the minimal UV-C erythema dose (MED) ascertained. Thereafter, the left and right medial buttock were irradiated with two 5-fold MED doses delivered to each site. The UV-C irradiation covered two circular skin spot of 1 cm2 at a distance of 4 cm. According to the manufacturer of the UV-C source (Somedic, Sweden), a maximum dose of 256 mJ/cm^2^ could be delivered. Dependent on their individual MED, six panellists were irradiated with 256 mJ/cm2, seven subjects received 181 mJ/cm^2^, and 3 subjects had doses of 128 mJ/cm^2^.

### Experimental Protocol

The time course of UV-C induced inflammatory skin sensitization and gene expression changes were investigated 3–6–24 h after UV-C treatment. A non-irradiated medial buttock site served as reference control.

### Assessment of Skin Sensitization

*Dynamic mechanical impact pain* was investigated by means of a cylindrical plastic bullet accelerated pneumatically in an 8 cm guiding barrel towards the skin surface [33]. The computer-controlled bullet velocity was set at 12 m/s in order to achieve sufficient nociceptor activation and the magnitude of impact-induced pain was rated on a VAS (15 cm length) displayed vertically on a computer screen with the endpoints “no pain” (bottom) and “worst pain” (top). Mechanical impact stimuli were administered in triplicate at 0.5 Hz intervals.

*Supra-threshold heat stimulation* was induced by delivering 48°C continuously for 5 sec via a surface thermode covering a 1 cm^2^ skin area (SENSELab, Somedic, Sweden). Peak maximum heat pain was recorded by VAS.

*Skin erythema upon mechanical and thermal stimuli* was investigated by laser Doppler imaging (Moor LDI, Axminster, UK), as an objective readout for activation of mechanically insensitive nociceptors. At a scan speed of 4 ms/pixel superficial skin blood flow was assessed in a rectangular skin area of 25 cm^2^ requiring 30 sec for image capture. After baseline blood flow measurement, mechanical or thermal stimuli were administered and axon reflex flare responses recorded for 5 min. Off-line analyses of the erythema reaction were performed by subtraction of the baseline images and calculation of the area of all pixels exceeding the 2-fold standard deviation of median baseline fluxes [34].

*Static mechanical sensitivity* was investigated at peak hyperalgesia 24 h post UV-C irradiation only. Delivering a force of 14 mN, a Semmes-Weinstein monofilament (Touch-Test™ - Sensory Evaluator, US) was applied for 3 sec to the skin and subjects were requested to estimate intensity of the perception relative to the sensation induced upon stimulation with 260 mN on a numeric rating scale (NRS) with the endpoints 0 (no sensation) and 100 (sensation perceived upon 260 mN). Stimuli were repeated three times in UV-C and non-irradiated skin, respectively, and in randomized order. A stimulation with 260 mN to the lateral non-irradiated buttock site preceded each of the 14 mN test stimuli in order to facilitate the comparison to the reference NRS of 100. From each skin site (UV-C and non-irradiated) the average NRS-ratings were calculated.

*Static mechanical pain* was investigated at peak hyperalgesia 24 h post UV-C irradiation only. A custom made pin prick stimulator [35] delivering a force of 512 mN (tip diameter 0.2 mm) was applied for 3 sec to the UV-C and non-irradiated skin, respectively. Subjects were asked to rate their pain sensation on a VAS. Stimuli were repeated three times at both skin sites and in randomized order. The average pain rating recorded from the UV-C and control skin was calculated for analysis.

### Expression Patterns

After the above functional tests, 4 *skin punch biopsies* were sampled from each volunteer, 1 taken from non-irradiated skin serving as control, and 3 taken from the irradiated sites 3, 6, and 24 h after UV-exposure. Skin biopsies were obtained by punches (3 mm diameter, Biopsy Punch, Stiefel, Germany) after intradermal injection of 150 µl Mepivacainhydrochlorid 1% (AstraZeneca, Sweden). All skin samples were flash frozen instantly by placing them - epidermis site down - on dry ice. Biopsies were stored at −80°C until mRNA PCR analysis.

### RNA Isolation

Frozen skin biopsies were mechanically disrupted in RLT lysis buffer (Qiagen, Germany) supplemented with 1% 2-Mercaptoethanol using an Ultra-Turrax (Janke & Kunkel, IKA-Werk, Germany) tissue homogeniser. RNA isolation was performed with RNeasy® Mini Kit and a silica-membrane binding capacity of 100 µg RNA (Qiagen, Germany). A protocol developed for animal tissues and cells including DNase digestion during RNA purification was used. Quality control and quantification of RNA was performed on Agilent 2100 Bioanalyzer and RNA integrity numbers >7 were considered acceptable.

### Reverse Transcription

High Capacity RNA-to-cDNA Kit (Applied Biosystems, Sweden) was used for reverse transcription Volumes and temperatures were applied as suggested by the manufacturer. All reverse transcriptions were performed in duplicate with 450 ng total RNA in each reaction. Blank control reactions without reverse transcriptase (no RT controls) were made to enable monitoring genomic DNA (gDNA) contamination.

### Quantitative Polymerase Chain Reaction (qPCR)

All qPCR reactions were run on an Applied Biosystems 7900HT Real-Time PCR system with sequence detecting system (SDS) software version 2.4 (Applied Biosystems, Sweden). Default PCR temperatures and cycle number (40 cycles) were used on optical 384-well Reaction Plates equipped with MicroAmp™ Optical Adhesive Covers (Applied Biosystems). Samples from two subjects, however, were of poorer quality (RNA integrity number <7) and quantity than all other probes. Initial statistical evaluation identified those subjects as “outliers”, which therefore were discarded from the analysis.

### Reference Genes and TaqMan® Gene Expression Assays

A panel of potential reference genes was evaluated by performing qPCR on all skin biopsy cDNA samples and comparing treatment groups to find the most stable reference gene according to TaqMan® Endogenous Control Assays (Applied Biosystems). Beta-glucoronidase gene (GUSB, Applied Biosystems) showing low variability, high PCR efficiency and an expression level close to the expected expression levels of the target genes, was used as reference gene. The ability of all TaqMan® Gene Expression Assays (Applied Biosystems) to amplify genomic DNA was tested by performing qPCR on a dilution series of human high-quality genomic DNA (Clontech, France). TaqMan® Gene Expression Assays that amplified gDNA No RT controls were analysed, but contribution of gDNA contamination in RNA samples did not impact qPCR interpretation (data not shown).

### Controls and Quantification Calibrators

A pool of total human testis and fetal brain RNA (Clontech, France) was reverse transcribed, using the reagents mentioned above, to provide a standard quantification calibrator. Serial dilutions of this standard cDNA were used to determine PCR efficiency of all TaqMan® Gene Expression Assays and values <80% efficiency excluded from analysis. Control reactions with no template were included for all gene expression assays performed on all assay plates.

### Data Analysis

The standard curve method was used in the SDS2.4 software to calculate quantities of target and reference genes. Data were normalised by dividing target gene quantity with reference gene quantity. Average values of the duplicate reverse transcriptions were calculated. Prior to statistical analysis, data was log transformed. Values of non-irradiated skin biopsies were compared to post-treatment time points by t-tests within a repeated measures 1-way analysis of variance (ANOVA) using GraphPad Prism 5.0 software (GraphPad Software Inc., CA, US). The ratios of the target gene expression at post-treatment time points divided by the expression without treatment are depicted with 95% confidence intervals (fig. 3). Pearson’s correlation coefficient was calculated to identify data correlation between the expression levels of candidate genes analyzed 3, 6, and 24 h after UV-C irradiation and the parameters of sensitization (stimuli evoked “pain” and “erythema”) recorded at peak level 24 h after irradiation (fig. 4) with Bonferroni corrections controlling for multiple testing.

Heat hyperalgesia and mechanical sensitization to impact stimuli were evaluated by ANOVA with the grouping variables “UV-C/control skin“ and “time post irradiation”, followed by Fisher’s Least Significant Difference (LSD) post hoc test. Pain estimates to pin-prick stimuli were analyzed by Wilcoxon’s matched pairs test. Levels of significance were p<0.05 and data depicted as means ± SEM (fig. 1 and 2).
